# How to follow the guidelines, when the appropriate fluid is missing?

**DOI:** 10.1007/s00431-024-05514-6

**Published:** 2024-03-18

**Authors:** David W. Brossier, Isabelle Goyer, Claire Morice, Fahad Alsohime, Huw F. Mayberry, Florence Porcheret, Lyvonne N. Tume, Frederic V. Valla, on behalf of the European Society of Pediatric and Neonatal Intensive Care (ESPNIC) IVMFT group, Sophie Beldjilali, Fabrizio Chiusolo, Leonardo Costa, Capucine Didier, Stavroula Ilia, Nyandat L Joram, Corinne Jotterand Chaparro, Martin CJ Kneyber, Eva Kühlwein, Jorge Lopez, Jesus López-Herce, Luise V. Marino, Fortesa Mehmeti, Magdalena Mierzewska-Schmidt, MarIa Miñambres Rodríguez, Clémence Moullet, John V. Pappachan, Leonor Reis Boto, Shancy Rooze, Luregn J Schlapbach, Hakan Tekguc, Konstantinos Tziouvas, Sascha CAT Verbruggen

**Affiliations:** 1 CHU de Caen, Pediatric Intensive Care Unit, 14000 Caen, France; 2 Medical School, Université Caen Normandie, 14000 Caen, France; 3 Université de Lille, CHU Lille, ULR 2694 - METRICS: Évaluation des technologies de santé et des pratiques médicales, 59000 Lille, France; 4 CHU Sainte Justine Research Center, Montréal, Canada; 5 CHU de Caen, Department of Pharmacy, 14000 Caen, France; 6 Pediatric Intensive Care, University Hospital of Geneva, Geneva, Switzerland; 7 Pediatric Intensive Care, Pediatric Department, College of Medicine, King Saud University, Riyadh, Saudi Arabia; 8 Pediatric Intensive Care Unit, Alder Hey Childrens Hospital, Liverpool, UK; 9 Department of Pediatric Nephrology, CHU de Nantes, 44000 Nantes, France; 10 Faculty of Heath Social Care & Medicine, Edge Hill University, Ormskirk, UK; 11 Hospices Civils de Lyon, Pediatric Intensive Care, 69000 Lyon, France

**Keywords:** Intravenous fluids, Balanced fluids, Isotonic fluids

## Abstract

Intravenous maintenance fluid therapy (IV-MFT) is probably the most prescribed drug in paediatric hospital care. Recently paediatric societies have produced evidence-based practice guidelines that recommend the use of balanced isotonic fluid when prescribing IV-MFT in both acute and critical paediatric care. Unfortunately, the applicability of these guidelines could be called into question when a ready-to-use glucose-containing balanced isotonic fluid is not available. The main objective of this study was to describe the availability of glucose-containing balanced isotonic fluids in European and Middle Eastern paediatric acute and critical care settings. This work is an ancillary study of the survey dedicated to IV-MFT practices in the paediatric acute and critical care settings in Europe and Middle East, a cross-sectional electronic 27-item survey, emailed in April–May 2021 to paediatric critical care physicians across 34 European and Middle East countries. The survey was developed by an expert multi-professional panel within the European Society of Peadiatric and Neonatal Intensive Care (ESPNIC). Balanced isotonic fluid with glucose 5% was available for only 32/153 (21%) responders. Balanced isotonic fluid with glucose 5% was consistently available in the UK (90%) but not available in France, Greece, The Netherlands and Turkey.

*    Conclusion*: Ready-to-use isotonic balanced IV solutions containing glucose in sufficient amount exist but are inconsistently available throughout Europe. National and European Medication Safety Incentives should guarantee the availability of the most appropriate and safest IV-MFT solution for all children.

**Table:** 

**What is Known:** *• Intravenous maintenance fluid therapy (IV-MFT) is probably the most prescribed drug in paediatric hospital care.* *• Balanced isotonic fluid is recommended when prescribing IV-MFT in both acute and critical paediatric care.*	
**What is New:** *• Balanced isotonic fluid with glucose 5% is available for less than 25% of the prescribers in Europe and the Middle East. Availability of balanced isotonic fluid with glucose 5% varies from one country to another but can also be inconsistent within the same country.* *• Clinicians who have access to a ready-to-use balanced isotonic fluid with glucose 5% are more likely to consider its use than clinicians who do not have access to such an IV solution.*

**Supplementary Information:**

The online version contains supplementary material available at 10.1007/s00431-024-05514-6.

## Introduction

Intravenous maintenance fluid therapy (IV-MFT) is probably the most prescribed drug in paediatric hospital care [1]. It has been used for more than a century and yet, it is only recently that paediatric societies have produced evidence-based practice guidelines to guide the use of IV fluids in clinical practice [2, 3]. These guidelines recommend the use of balanced isotonic fluid when prescribing IV-MFT in both acute and critical paediatric care. Those recommendations were based on the fact that balanced isotonic fluids were less likely to cause hyponatremia and metabolic hyperchloremic acidosis, which have been associated with several severe, potentially deadly, complications in the pediatric intensive care unit (PICU), such as neurological impairment, kidney injury or organ dysfunction [4]. Besides, balanced solutions have also been shown to reduce the length of both PICU and hospital stay [3]. It is also recommended to provide the appropriate amounts of potassium and glucose to prevent children from presenting hypokalaemia and hypoglycaemia [2, 3]. However, even though two international paediatric societies (the American Academic of Pediatrics (AAP) [2] and the European Society of Paediatric and Neonatal Intensive Care (ESPNIC) [3]) are to be commended for these long-awaited guidelines, the applicability and implementation of these guidelines are threatened by a lack of these fluids available. During the last decade, the growing interest in the use of balanced crystalloids to prevent patients from developing clinical complications and mortality related to hyperchloremia and metabolic acidosis has been associated with a growing availability of balanced IV fluids [5]. Unfortunately, amongst this variety of available balanced IV fluids, very few, or even none in certain countries, contain glucose. However, glucose content is fundamental for paediatric IV-MFT. In 2022, Morice et al. showed that the absence of glucose in the solution was the main reason for not prescribing a balanced fluid by 29.4% of the respondents [1]. The main objective of this study was to describe the availability of glucose-containing balanced isotonic fluids in European and Middle Eastern paediatric acute and critical care settings, performing a complementary analysis of the Morice et al. [1] survey. The secondary objective was to evaluate the impact of the absence of paediatric appropriate ready-to-use fluids on IV-MFT declarative practice.

## Materials and method

This work was an ancillary study of the survey dedicated to IV-MFT practice in the paediatric acute and critical care settings in 35 countries in Europe and Middle East [1]. The study design, the included population and the survey instrument development, content and data collection have previously been published [1]. This survey was designed to collect a single response per centre.

### Data analysis

Data were analysed according to the country of the responders and according to the availability of balanced isotonic fluids, with or without glucose 5%. The data analysis was focused on the questions related to the use of balanced fluids (Q13, 14, 15, 16) and fluid choices (Q17, 18, 19, 20).

We used a summative score to summarize the results from Likert scale questions for each participant. Variables distributions were assessed by the Shapiro–Wilk comparison test and continuous variables were presented as median (min-max). Categorical variables were presented as number (percentage). Comparisons between both groups were made by a Mann–Whitney *U* test or a Kruskal-Wallis’s test for continuous variables as appropriate and by a chi-square test with Monte Carlo simulation with 2000 replicates for categorical variables. The level of statistical significance was set at *p* < 0.05. Statistical analyses were performed using open-access R software (Version 4.2.1; R Foundation for Statistical Computing, Vienna, Austria). Ethical approval was obtained from the Caen-France institutional review board (reference number 2474).

## Results

### Participants’ characteristics

Participants’ characteristics were presented in [1]. The response rate related to contacted centres was 63%, with 153 centres represented, over 240 contacted. The responses represented 35 (82%) of the 43 countries surveyed. One participant was excluded for practicing in Australia.

### Fluid availability according to country

Fluid availability according to the country of the responders is presented in Table 1. Balanced isotonic fluid with glucose 5% was declared available for only 32 (21%) responders. Balanced isotonic fluid with glucose 5% was consistently available only in the UK (90%) and totally absent from France, Greece, The Netherlands and Turkey. The most widely available fluids were balanced (93.5%) and unbalanced (87.6%) isotonic fluids without glucose.

**Table 1:** Fluid availability in Europe and Middle East

	Total	Belgium	France	Germany	Greece	Italy	Poland	Portugal	Spain	Switzerland	The Netherlands	Turkey	United Kingdom	Others	*p*
	*n* = 153	*n* = 7	*n* = 17	*n* = 10	*n* = 5	*n* = 10	*n* = 9	*n* = 7	*n* = 19	*n* = 11	*n* = 5	*n* = 9	*n* = 10	*n* = 34	
Balanced isotonic fluid	143 (93.5%)	6 (85.7%)	15 (88.2%)	8 (80%)	5 (100%)	10 (100%)	9 (100%)	7 (100%)	17 (89.5%)	11 (100%)	5 (100%)	8 (88.9%)	10 (100%)	32 (94.1%)	0.68
Balanced isotonic fluid with glucose 5%	32 (21.0%)	4 (57.1%)	0	2 (20.0%)	0	1 (10.0%)	1 (11.1%)	3 (42.9%)	4 (21.1%)	3 (27.3%)	0	0	9 (90%)	5 (14.7%)	< 0.0001
Balanced isotonic fluid with glucose 1%	23 (15.0%)	0	8 (47.1%)	3 (30%)	0	0	6 (66.7%)	0	1 (5.3%)	4 (36.4%)	0	0	0	1 (2.9%)	< 0.0001
Balanced hypotonic fluid with glucose 5%	4 (2.6%)	1 (14.3%)	0	0	0	2 (20%)	0	0	0	0	0	0	0	1 (2.9%)	0.10
Unbalanced isotonic fluid	134 (87.6%)	6 (85.7%)	14 (82.4%)	6 (60%)	5 (100%)	10 (100%)	5 (55.6%)	7 (100%)	17 (89.5%)	10 (90.9%)	4 (80%)	9 (100%)	10 (100%)	31 (91.2%)	0.03
Unbalanced isotonic fluid with glucose 5%	7 (4.6%)	1 (14.3%)	0	0	0	0	0	1 (14.3%)	4 (21.1%)	0	0	0	1 (10%)	0	0.06
Unbalanced hypotonic fluid with glucose 5%	114 (74.5%)	6 (85.7%)	17 (100%)	6 (60%)	4 (80%)	4 (40%)	3 (33.3%)	7 (100%)	14 (73.7%)	7 (63.6%)	4 (80%)	9 (100%)	9 (90%)	24 (70.6%)	0.001

Balanced isotonic fluids are as follows: Ringer’s lactate, Ringer’s acetate, Hartamann’s solution, Plasmalyte, Isolyte E and S, Normosol R, Isofundine, Sterofundine, Ringerfundine, Optilyte

Balanced isotonic fluids with glucose 5% are as follows: PlasmalyteG5, Sterofundine VG5

Balanced isotonic fluids with glucose 1% are as follows: Isopedia, Benelyte

Balanced hypotonic fluids with glucose 5% are as follows: Normosol M; Sterofundine HEG 5; Isolyte G, M and P

Unbalanced isotonic fluid is as follows: NaCl 0.9%

Unbalanced isotonic fluid with glucose 5% is as follows: G5NaCl 0.9%

Unbalanced hypotonic fluids with glucose 5% are as follows: Glucidion, Osmotan, Bionolyte, Polyionique, Dextrion

^a^*n* refers to the number of centers

### Impact of country on prescriptions' practices

Responders’ consideration for the importance of balanced fluids varies considerably between countries in both conventional and critical care unit (SDC 1). Prescription practices varied considerably between countries (SDC 1). Balanced isotonic fluid was considered in 45.0% of the clinical situations (from 6.5% in Greece to 83.3% in Poland) and unbalanced isotonic fluid in 42.8% (from 11.1% in Poland to 78.5% in Turkey). Hypotonic unbalanced fluid was considered in 10.5% of the clinical situations (from 0% in the UK to 30% in Greece). It was consistently the less prescribed fluid, except in France and in Greece, where it was prescribed more than balanced isotonic fluid.

### Impact of fluid availability on prescriptions’ practices

Among the 32 responders who declared having access to a balanced isotonic fluid with glucose 5%, 23 (71.9%) reported that balanced isotonic fluid should be always considered vs 42/121 (34.7%) (*p* < 0.001) in the case of unavailability of a balanced isotonic fluid with glucose 5% (SDC 2). The availability of a balanced isotonic fluid with glucose 5% was systematically and significantly associated with a preference for prescribing this fluid over unbalanced isotonic or hypotonic crystalloids, notwithstanding the clinical situation studied (SDC 2).

## Discussion

Performing a complementary analysis on the declarative data of Morice et al. survey [1], focusing on the declared type of available IV fluids, we have realised that only 21% of responders have access to a commercialized balanced isotonic fluid containing 5% glucose, which is considered as the current recommended IV fluid for paediatric IV-MFT. We have shown that the availability of such a solution varies from one country to another but can also be inconsistent within the same country. In addition, we have observed that the availability of a balanced isotonic fluid with 5% glucose was associated with a higher declarative use of balanced isotonic fluid in almost all the assessed clinical situations. This inconsistency regarding the availability of these ready-made balanced solutions is a significant barrier to the implementation of the recent ESPNIC IV-MFT guidelines into clinical practices and could explain the obsolete but still current use of hypotonic IV fluids [1, 6]. This should be reassessed once a specific model for disseminating these guidelines in clinical practice has been implemented.

**Fig. 1:**
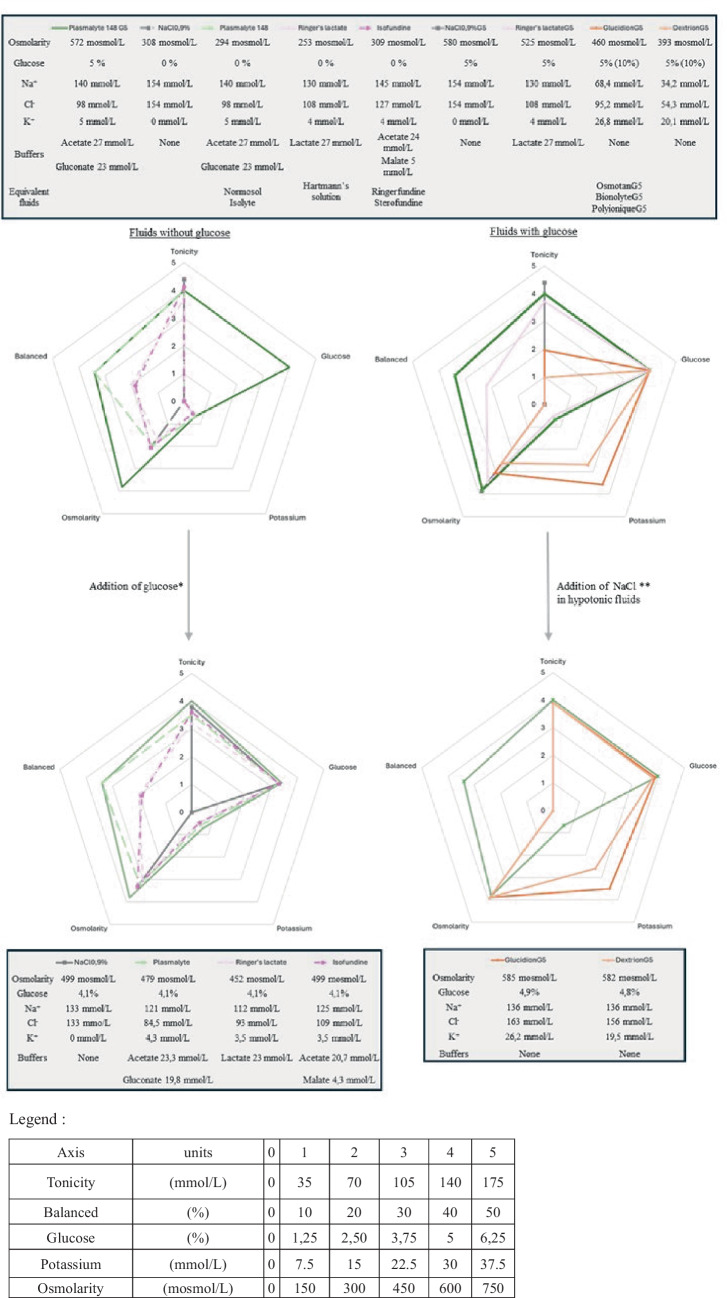
Characteristics of maintenance intravenous fluid solution, adapted from [6]. *Adjunction of glucose is 80 mL of glucose 30% per 500 mL. **Adjunction of NaCl is 10 mL of NaCl (2 g/10 mL) per 500 mL in Glucidion or equivalent, and 15 mL of NaCl (2 g/10 mL) per 500 mL in Dextrion or equivalent. ***Balanced was assessed on the basis of the percentage of buffers relative to the total concentration of anions. Plasmalyte 148 G5 is presented in every figure as the only available reference fluid in Europe

In the absence of a ready-to-use appropriate IV fluid for children, local compounding to make solutions that comply with the recommendations is often required (Fig. 1). Such manipulations give rise to significant patient risks regarding the uncertainty in physico-chemical stability, microbial contamination, prescription and preparation errors while manipulating electrolytes, as well as alterations of the tonicity and/or the balanced nature of the original fluid [7]. Some clinicians have considered using paediatric IV fluids marketed for the peri-operative period as alternatives that are balanced isotonic fluids with 1% glucose. ISOPEDIA^©^ (FRESENIUS KABI FRANCE) and BENELYTE^©^ (FRESENIUS KABI POLSKA) are the only balanced isotonic glucose-containing crystalloids available in many European countries. Their marketing authorisation was obtained in 2017, based on perioperative IV-MFT guidelines in children, which recommended a 1 to 2.5% glucose concentration [8]. However, this glucose-containing fluid is probably not appropriate for use outside of the perioperative setting, as they provide insufficient amount of glucose. No clear consensus exists on the optimal glucose concentration for paediatric IV-MFT. In the general paediatric setting, 5% glucose concentration solutions are common and recommended by some medical societies [9] probably based on Holliday and Segar guidance [6]. Likewise, adult guidelines suggest considering a daily glucose intake of 1 to 1.5 g/kg/day to prevent fasting ketonemia [10]. We consider that isotonic balanced solutions which would provide different ranges of glucose (from 1 to 10%) should be favoured and made readily available on the market to ensure safe IV fluid therapy for children. In addition, as the insufficient amount of potassium in some balanced fluids has been called into question and may contribute to impairing the applicability of the guidelines, those fluids should be available with a sufficient amount of potassium for use in standard paediatric IV maintenance therapy [11]. Specific considerations should be made regarding potassium content when bolus fluids are used or in case of renal failure. Finally, consideration should be given to cost and packaging. If these recommendations are to be applicable worldwide, including in low- and middle-income countries, the recommended fluids must be available at a reasonable price [12]. In addition, to overcoming the wide variability in patient characteristics encountered in paediatric practice, the recommended fluids should be available in a range of packaging formats, in order to reduce waste as well as the environmental footprint of plastic packaging [13].

The limitations inherent to the original survey were presented in [1]. This study’s specific limitations mainly lie within the fact that the survey was not originally dedicated to determining the different fluid availability. It is therefore difficult to confirm that unavailability of the appropriate fluid in responding centres of one country reflects the absence of marketing of the fluid within the country or the simple lack of product referencing in the responding centre (due to cost issues or poor regard to the necessity of the product). This study was not designed to identify potential stakeholders in the availability of balanced fluids.

## Conclusion

Ready-to-use isotonic balanced IV solutions containing glucose in sufficient amounts exist but are inconsistently available throughout Europe. National and European Medication Safety Incentives should guarantee the availability of the most appropriate and safest IV-MFT solution for all children. Our expert group is calling for the rapid commercialization of appropriate solutions worldwide.

### Supplementary Information

Below is the link to the electronic supplementary material.

Supplementary file1 (DOCX 46 KB)

Supplementary file2 (DOCX 43 KB)

## Data Availability

No datasets were generated or analysed during the current study.

## Abbreviations

AAP: American Academy of Pediatrics
ESPNIC: European Society of Pediatric and Neonatal Intensive Care
IV: Intravenous
IV-MFT: Intravenous maintenance fluid therapy
PICU: Paediatric intensive care unit
