# Supplementary material for: How to follow the guidelines, when the appropriate fluid is missing?
Source: Eur J Pediatr. 2024 Mar 18;183(6):2797–803. doi: 10.1007/s00431-024-05514-6 (PMC11098858; doi:10.1007/s00431-024-05514-6)
Supplement: Supplementary file 1 — Supplementary file1 (DOCX 46 KB) [file 431_2024_5514_MOESM1_ESM.docx]

Supplemental digital content 1: Impact of the country on prescription practices

|  | Total | Belgium | France | Germany | Greece | Italy | Poland | Portugal | Spain | Switzerland | The Netherlands | Turkey | United Kingdom | Others | p |
| --- | --- | --- | --- | --- | --- | --- | --- | --- | --- | --- | --- | --- | --- | --- | --- |
|  | n=153 | n=7 | n=17 | n=10 | n=5 | n=10 | n=9 | n=7 | n=19 | n=11 | n=5 | n=9 | n=10 | n=34 |  |
| Importance of prescribing balanced isotonic fluid (0 to 10 scale) | | | | | | | | | | | | | | | |
| In conventional unit |  | 7.5  [6-9] | 5  [0-10] | 7.5  [2-10] | 5  [0 -7] | 8.5  [4-10] | 10  [4-10] | 8  [6-10] | 7.5  [0-10] | 8  [5-10] | 8  [3-9] | 6  [3-10] | 8.5  [2-10] | 7.5  [1-10] | < 0.01 |
| In critical care unit |  | 9  [7-10] | 8  [1-10] | 9.5  [5-10] | 6  [0 -7] | 10  [5-10] | 10  [4-10] | 9  [8-10] | 8  [0-10] | 8  [5-10] | 9  [3-9] | 7  [3-10] | 9.5  [2-10] | 9  [3-10] | 0.02 |
| Proportion of physicians considering balanced solution as a standard of care | | | | | | | | | | | | | | | |
| Always | 65  (42.5%) | 4  (57.1%) | 2  (11.8%) | 8  (80.0%) | 1  (20.0%) | 6  (60.0%) | 6  (66.7%) | 2  (28.6%) | 5  (26.3%) | 7  (63.6%) | 1  (20.0%) | 1  (11.1%) | 9  (90.0%) | 13  (38.2%) | < 0.001 |
| Limiting factors regarding balanced isotonic fluid prescription | | | | | | | | | | | | | | | |
| Unavailability of the fluid | 45  (29.4%) | 1  (14.3%) | 6  (35.3%) | 2  (20.0%) | 2  (40.0%) | 1  (10.0%) | 3  (33.3%) | 4  (57.1%) | 2  (10.5%) | 2  (18.2%) | 0 | 5  (55.6%) | 3  (30.0%) | 14  (41.2%) | 0.14 |
| Cost of the fluid | 26 (17.0%) | 2  (28.6%) | 3  (17.6%) | 0 | 2  (40.0%) | 0 | 0 | 2  (28.6%) | 3  (15.8%) | 1  (9.1%) | 0 | 4  (44.4%) | 3  (30.0%) | 6  (17.6%) | 0.16 |
| Ready to use fluid | 50  (32.7%) | 4  (57.1%) | 7  (41.2%) | 4  (40.0%) | 2  (40.0%) | 2  (20.0%) | 3  (33.3%) | 2  (28.6%) | 3  (15.8%) | 2  (18.2%) | 0 | 5  (55.6%) | 4  (40.0%) | 12  (35.3%) | 0.47 |
| Type of fluids according to clinical situation | | | | | | | | | | | | | | | |
| *Viral gastroenteritis not tolerating oral rehydration, with normal natremia (137mmol/L); 5 months old* | | | | | | | | | | | | | | | 0.046 |
| Unbalanced Hypotonic Fluid | 10  (6.5%) | 0 | 3  (17.6%) | 1  (10.0%) | 1  (20.0%) | 0 | 0 | 0 | 1  (5.3%) | 1  (9.1%) | 1  (20.0%) | 0 | 0 | 2  (5.9%) |  |
| Unbalanced Isotonic Fluid | 71  (46.4%) | 4  (57.1%) | 10  (58.8%) | 2  (20.0%) | 3  (60.0%) | 1  (10.0%) | 1  (11.1%) | 4  (57.1%) | 15  (78.9%) | 3  (27.3%) | 1  (20.0%) | 8  (88.9%) | 4  (40.0%) | 15  (44.1%) |  |
| Balanced Isotonic Fluid | 71  (46.4%) | 3  (42.9%) | 3  (17.6%) | 7  (70.0%) | 1  (20.0%) | 9  (90.0%) | 8  (88.9%) | 3  (42.9%) | 3  (15.8%) | 7  (63.6%) | 3  (60.0%) | 1  (11.1%) | 6  (60.0%) | 17  (50.0%) |  |
| *Viral gastroenteritis not tolerating oral rehydration, with normal natremia (137mmol/L); 12 years old* | | | | | | | | | | | | | | | 0.0045 |
| Unbalanced Hypotonic Fluid | 8  (5.2%) | 1  (14.3%) | 3  (17.6%) | 0 | 1  (20.0%) | 0 | 0 | 0 | 0 | 1  (9.1%) | 1  (20.0%) | 0 | 0 | 1  (2.9%) |  |
| Unbalanced Isotonic Fluid | 69  (45.1%) | 3  (42.9%) | 9  (52.9%) | 1  (10.0%) | 3  (60.0%) | 2  (20.0%) | 1  (11.1%) | 5  (71.4%) | 16  (84.2%) | 3  (27.3%) | 0 | 8  (88.9%) | 4  (40.0%) | 14  (41.2%) |  |
| Balanced Isotonic Fluid | 75  (49.0%) | 3  (42.9%) | 4  (23.5%) | 9  (90.0%) | 1  (20.0%) | 8  (80.0%) | 8  (88.9%) | 2  (28.6%) | 3  (15.8%) | 7  (63.6%) | 4  (80.0%) | 1  (11.1%) | 6  (60.0%) | 19  (55.9%) |  |
| *Viral gastroenteritis not tolerating oral rehydration, with hypernatremia (> 149 mmol/L); 5 months old* | | | | | | | | | | | | | | | 0.14 |
| Unbalanced Hypotonic Fluid | 41  (26.8%) | 1  (14.3%) | 4  (23.5%) | 2  (20.0%) | 3  (60.0%) | 2  (20.0%) | 4  (44.4%) | 3  (42.9%) | 3  (15.8%) | 2  (18.2) | 2  (40.0%) | 4  (44.4%) | 0 | 11  (32.4%) |  |
| Unbalanced Isotonic Fluid | 35  (22.9%) | 1  (14.3%) | 5  (29.4%) | 0 | 1  (20.0%) | 1  (10.0%) | 0 | 1  (14.3%) | 11  (57.9%) | 1  (9.1) | 1  (20.0%) | 3  (33.3%) | 2  (20.0%) | 8  (23.5%) |  |
| Balanced Isotonic Fluid | 76  (49.7%) | 5  (71.4%) | 7  (41.2%) | 8  (80.0%) | 1  (20.0%) | 7  (70.0%) | 5  (55.6%) | 3  (42.9%) | 5  (26.3%) | 8  (72.7) | 2  (40.0%) | 2  (22.2%) | 8  (80.0%) | 15  (44.1%) |  |
| *Viral gastroenteritis not tolerating oral rehydration, with hypernatremia (> 149 mmol/L); 12 years old* | | | | | | | | | | | | | | | 0.045 |
| Unbalanced Hypotonic Fluid | 38  (24.8%) | 2  (28.6%) | 4  (23.5%) | 2  (20.0%) | 3  (60.0%) | 2  (20.0%) | 4  (44.4%) | 2  (28.6%) | 2  (10.5%) | 1  (9.1) | 2  (40.0%) | 2  (22.2%) | 0 | 12  (35.3%) |  |
| Unbalanced Isotonic Fluid | 34  (22.2%) | 0 | 3  (17.6%) | 1  (10.0%) | 1  (20.0%) | 1  (10.0%) | 0 | 1  (14.3%) | 12  (63.2%) | 2  (18.2) | 0 | 5  (55.6%) | 2  (20.0%) | 6  (17.6%) |  |
| Balanced Isotonic Fluid | 80  (52.3%) | 5  (71.4%) | 9  (52.9%) | 7  (70.0%) | 1  (20.0%) | 7  (70.0%) | 5  (55.6%) | 4  (57.1%) | 5  (26.3%) | 8  (72.7) | 3  (60.0%) | 2  (22.2%) | 8  (80.0%) | 16  (47.1%) |  |
| *Status epilepticus with anormal level of consciousness; 5 months old* | | | | | | | | | | | | | | | 0.015 |
| Unbalanced Hypotonic Fluid | 6  (3.9%) | 0 | 1  (5.9%) | 1  (10.0%) | 1  (20.0%) | 1  (10.0%) | 0 | 0 | 0 | 0 | 1  (20.0%) | 0 | 0 | 1  (2.9%) |  |
| Unbalanced Isotonic Fluid | 84  (54.9%) | 3  (42.9%) | 13  (76.5%) | 2  (20.0%) | 4  (80.0%) | 1  (10.0%) | 1  (11.1%) | 5  (71.4%) | 16  (84.2%) | 6  (54.5%) | 2  (40.0%) | 8  (88.9%) | 4  (40.0%) | 19  (55.9%) |  |
| Balanced Isotonic Fluid | 62  (40.5%) | 4  (57.1%) | 2  (11.8%) | 7  (70.0%) | 0 | 8  (80.0%) | 8  (88.9%) | 2  (28.6%) | 3  (15.8%) | 5  (45.5%) | 2  (40.0%) | 1  (11.1%) | 6  (60.0%) | 14  (41.2%) |  |
| *Status epilepticus with anormal level of consciousness; 12 years old* | | | | | | | | | | | | | | | 0.003 |
| Unbalanced Hypotonic Fluid | 4  (2.6%) | 0 | 1  (5.9%) | 1  (10.0%) | 1  (20.0%) | 0 | 0 | 0 | 0 | 0 | 1  (20.0%) | 0 | 0 | 0 |  |
| Unbalanced Isotonic Fluid | 77  (50.3%) | 3  (42.9%) | 11  (64.7%) | 1  (10.0%) | 4  (80.0%) | 1  (10.0%) | 1  (11.1%) | 2  (28.6%) | 17  (89.5%) | 6  (54.5%) | 1  (20.0%) | 8  (88.9%) | 4  (40.0%) | 18  (52.9%) |  |
| Balanced Isotonic Fluid | 71  (46.4%) | 4  (57.1%) | 4  (23.5%) | 8  (80.0%) | 0 | 9  (90.0%) | 8  (88.9%) | 5  (71.4%) | 2  (10.5%) | 5  (45.5%) | 3  (60.0%) | 1  (11.1%) | 6  (60.0%) | 16  (47.1%) |  |
| *Severe diabetic Keto-acidosis; 6 years old* | | | | | | | | | | | | | | | 0.026 |
| Unbalanced Hypotonic Fluid | 8  (5.2%) | 0 | 3  (17.6%) | 0 | 0 | 0 | 0 | 0 | 3  (15.8%) | 0 | 1  (20.0%) | 0 | 0 | 1  (2.9%) |  |
| Unbalanced Isotonic Fluid | 81  (52.9%) | 4  (57.1%) | 10  (58.8%) | 4  (40.0%) | 4  (80.0%) | 5  (50.0%) | 1  (11.1%) | 4  (57.1%) | 12  (63.2%) | 5  (45.5%) | 2  (40.0%) | 8  (88.9%) | 5  (50.0%) | 17  (50.0%) |  |
| Balanced Isotonic Fluid | 62  (40.5%) | 3  (42.9%) | 3  (17.6%) | 6  (60.0%) | 0 | 5  (50.0%) | 8  (88.9%) | 3  (42.9%) | 4  (21.1%) | 6  (54.5%) | 2  (40.0%) | 1  (11.1%) | 5  (50.0%) | 16  (47.1%) |  |
| *Bronchiolitis with persistent respiratory distress under non-invasive ventilatory support; 7 days old* | | | | | | | | | | | | | | | 0.008 |
| Unbalanced Hypotonic Fluid | 31  (20.3%) | 0 | 5  (29.4%) | 2  (20.0%) | 2  (40%) | 1  (10%) | 0 | 2  (28.6%) | 5  (26.3%) | 3  (27.3%) | 2  (40.0%) | 5  (55.6%) | 0 | 4  (11.8%) |  |
| Unbalanced Isotonic Fluid | 63  (41.2%) | 3  (42.9%) | 10  (58.8%) | 2  (20.0%) | 3  (60%) | 1  (10%) | 1  (11.1%) | 4  (57.1%) | 12  (63.2%) | 1  (9.1) | 2  (40.0%) | 3  (33.3%) | 4  (40.0%) | 17  (50.0%) |  |
| Balanced Isotonic Fluid | 58  (37.9%) | 4  (57.1%) | 1  (5.9%) | 6  (60.0%) | 0 | 8  (80%) | 8  (88.9%) | 1  (14.3%) | 2  (10.5%) | 7  (63.6%) | 1  (20.0%) | 1  (11.1%) | 6  (60.0%) | 13  (38.2%) |  |
| *Bronchiolitis with persistent respiratory distress under non-invasive ventilatory support; 5 months old* | | | | | | | | | | | | | | | 0.003 |
| Unbalanced Hypotonic Fluid | 20  (13.1%) | 0 | 5  (29.4%) | 2  (20.0%) | 2  (40%) | 1  (10%) | 0 | 2  (28.6%) | 1  (5.3%) | 2  (18.2) | 1  (20.0%) | 1  (11.1%) | 0 | 3  (8.8%) |  |
| Unbalanced Isotonic Fluid | 71  (46.4%) | 3  (42.9%) | 10  (58.8%) | 2  (20.0%) | 3  (60%) | 1  (10%) | 1  (11.1%) | 2  (28.6%) | 16  (84.2%) | 2  (18.2) | 2  (40.0%) | 7  (77.8%) | 4  (40.0%) | 18  (52.9%) |  |
| Balanced Isotonic Fluid | 61  (39.9%) | 4  (57.1%) | 1  (5.9%) | 6  (60.0%) | 0 | 8  (80%) | 8  (88.9%) | 3  (42.9%) | 2  (10.5%) | 7  (63.6%) | 2  (40.0%) | 1  (11.1%) | 6  (60.0%) | 13  (38.2%) |  |
| *Pneumonia with persistent respiratory distress under non-invasive ventilatory support; 12 years old* | | | | | | | | | | | | | | | 0.0005 |
| Unbalanced Hypotonic Fluid | 13  (8.5%) | 0 | 4  (23.5%) | 1  (10.0%) | 2  (40.0%) | 0 | 0 | 2  (28.6%) | 0 | 2  (18.2) | 0 | 0 | 0 | 2  (5.9%) |  |
| Unbalanced Isotonic Fluid | 70  (45.8%) | 2  (28.6%) | 10  (58.8%) | 1  (10.0%) | 2  (40.0%) | 1  (10.0%) | 1  (11.1%) | 2  (28.6%) | 17  (89.5%) | 2  (18.2) | 1  (20.0%) | 9  (100%) | 4  (40.0%) | 18  (52.9%) |  |
| Balanced Isotonic Fluid | 69  (45.1%) | 5  71.4%) | 2  (11.8%) | 8  (80.0%) | 1  (20.0%) | 9  (90.0%) | 8  (88.9%) | 3  (42.9%) | 2  (10.5%) | 7  (63.6%) | 4  (80.0%) | 0 | 6  (60.0%) | 14  (41.2%) |  |
| *24 hours post-appendectomy monitoring, nil by mouth; 5 months old* | | | | | | | | | | | | | | | 0.0015 |
| Unbalanced Hypotonic Fluid | 16  (10.5%) | 0 | 6  (35.3%) | 1  (10.0%) | 2  (40.0%) | 1  (10.0%) | 0 | 2  (28.6%) | 1  (5.3%) | 1  (9.1%) | 1  (20.0%) | 0 | 0 | 1  (2.9%) |  |
| Unbalanced Isotonic Fluid | 63  (41.2%) | 3  (42.9%) | 8  (47.1%) | 1  (10.0%) | 3  (60.0%) | 2  (20.0%) | 1  (11.1%) | 2  (28.6%) | 15  (78.9%) | 3  (27.3%) | 1  (20.0%) | 8  (88.9%) | 3  (30.0%) | 13  (38.2%) |  |
| Balanced Isotonic Fluid | 70  (45.8%) | 4  (57.1%) | 2  (11.8%) | 8  (80.0%) | 0 | 7  (70.0%) | 8  (88.9%) | 3  (42.9%) | 2  (10.5%) | 7  (63.6%) | 3  (60.0%) | 1  (11.1%) | 7  (70.0%) | 18  (52.9%) |  |
| *24 hours post-appendectomy monitoring, nil by mouth; 12 years old* | | | | | | | | | | | | | | | 0.0015 |
| Unbalanced Hypotonic Fluid | 11  (7.2%) | 0 | 4  (23.5%) | 1  (10.0%) | 2  (40.0%) | 0 | 0 | 2  (28.6%) | 0 | 1  (9.1%) | 1  (20.0%) | 0 | 0 | 0 |  |
| Unbalanced Isotonic Fluid | 63  (41.2%) | 2  (28.6%) | 7  (41.2%) | 1  (10.0%) | 3  (60.0%) | 2  (20.0%) | 1  (11.1%) | 3  (42.9%) | 16  (84.2%) | 3  (27.3%) | 0 | 8  (88.9%) | 3  (30.0%) | 14  (41.2%) |  |
| Balanced Isotonic Fluid | 76  (49.7%) | 5  71.4%) | 5  (29.4%) | 8  (80.0%) | 0 | 8  (80.0%) | 8  (88.9%) | 2  (28.6%) | 2  (10.5%) | 7  (63.6%) | 4  (80.0%) | 1  (11.1%) | 7  (70.0%) | 19  (55.9%) |  |
| *Acute Respiratory Distress Syndrome under invasive ventilatory support; 5 months old* | | | | | | | | | | | | | | | 0.0040 |
| Unbalanced Hypotonic Fluid | 14  (9.2%) | 0 | 5  (29.4%) | 0 | 2  (40.0%) | 1  (10.0%) | 0 | 1  (14.3%) | 0 | 2  (18.2) | 0 | 1  (11.1%) | 0 | 2  (5.9%) |  |
| Unbalanced Isotonic Fluid | 69  (45.1%) | 2  (28.6%) | 9  (52.9%) | 2  (20.0%) | 3  (60.0%) | 1  (10.0%) | 2  (22.2%) | 4  (57.1%) | 16  (84.2%) | 2  (18.2) | 2  (40.0%) | 7  (77.8%) | 4  (40.0%) | 15  (44.1%) |  |
| Balanced Isotonic Fluid | 69  (45.1%) | 5  71.4%) | 2  (11.8%) | 8  (80.0%) | 0 | 8  (80.0%) | 7  (77.8%) | 2  (28.6%) | 3  (15.8%) | 7  (63.6%) | 3  (60.0%) | 1  (11.1%) | 6  (60.0%) | 17  (50.0%) |  |
| *Acute Respiratory Distress Syndrome under invasive ventilatory support; 12 years old* | | | | | | | | | | | | | | | 0.0005 |
| Unbalanced Hypotonic Fluid | 7  (4.6%) | 0 | 4  (23.5%) | 0 | 0 | 0 | 0 | 0 | 0 | 2  (18.2) | 0 | 0 | 0 | 1  (2.9%) |  |
| Unbalanced Isotonic Fluid | 70  (45.8%) | 1  (14.3%) | 8  (47.1%) | 1  (10.0%) | 5  (100.0%) | 2  (20.0%) | 2  (22.2%) | 5  (71.4%) | 17  (89.5%) | 2  (18.2) | 1  (20.0%) | 8  (88.9%) | 4  (40.0%) | 14  (41.2%) |  |
| Balanced Isotonic Fluid | 75  (49.0%) | 6  (85.7%) | 4  (23.5%) | 9  (90.0%) | 0 | 8  (80.0%) | 7  (77.8%) | 2  (28.6%) | 2  (10.5%) | 7  (63.6%) | 4  (80.0%) | 1  (11.1%) | 6  (60.0%) | 19  (55.9%) |  |
| *Post-traumatic brain injury with raised intra-cranial pressure; 14 years old* | | | | | | | | | | | | | | | 0.0015 |
| Unbalanced Hypotonic Fluid | 2  (1.3%) | 0 | 2  (11.8%) | 0 | 0 | 0 | 0 | 0 | 0 | 0 | 0 | 0 | 0 | 0 |  |
| Unbalanced Isotonic Fluid | 81  (52.9%) | 3  (42.9%) | 8  (47.1%) | 1  (10.0%) | 5  (100.0%) | 2  (20.0%) | 1  (11.1%) | 4  (57.1%) | 18  (94.7%) | 6  (54.5%) | 3  (60.0%) | 8  (88.9%) | 5  (50.0%) | 17  (50.0%) |  |
| Balanced Isotonic Fluid | 66  (43.1%) | 4  (57.1%) | 5  (29.4%) | 9  (90.0%) | 0 | 8  (80.0%) | 8  (88.9%) | 2  (28.6%) | 1  (5.3%) | 5  (45.5%) | 2  (40.0%) | 1  (11.1%) | 5  (50.0%) | 16  (47.1%) |  |
| *Post-cardiac surgery with bypass; 5 months old* | | | | | | | | | | | | | | | 0.0005 |
| Unbalanced Hypotonic Fluid | 20  (13.1%) | 0 | 7  (41.2%) | 0 | 1  (20.0%) | 1  (10.0%) | 0 | 1  (14.3%) | 2  (10.5%) | 3  (27.3%) | 1  (20.0%) | 1  (11.1%) | 0 | 3  (8.8%) |  |
| Unbalanced Isotonic Fluid | 50  (32.7%) | 2  (28.6%) | 6  (35.3%) | 0 | 4  (80.0%) | 1  (10.0%) | 1  (11.1%) | 2  (28.6%) | 11  (57.9%) | 1  (9.1) | 1  (20.0%) | 7  (77.8%) | 2  (20.0%) | 12  (35.3%) |  |
| Balanced Isotonic Fluid | 65  (42.5%) | 5  71.4%) | 1  (5.9%) | 8  (80.0%) | 0 | 7  (70.0%) | 8  (88.9%) | 1  (14.3%) | 2  (10.5%) | 7  (63.6%) | 3  (60.0%) | 1  (11.1%) | 6  (60.0%) | 16  (47.1%) |  |
| *All situations* | | | | | | | | | | | | | | | 0.0005 |
| Unbalanced Hypotonic Fluid | 256  (10.5%) | 6  (5.4%) | 63  (23.2%) | 16  (10.0%) | 24  (30%) | 10  (6.3%) | 8  (5.6%) | 17  (15.2%) | 18  (5.9%) | 21  (11.9%) | 15  (18.8%) | 14  (9.7%) | 0 | 44  (8.1%) |  |
| Unbalanced Isotonic Fluid | 1048  (42.8%) | 37  (33.0%) | 135  (49.6%) | 21  (13.1%) | 50  (62.5%) | 26  (16.3%) | 16  (11.1%) | 53  (47.3%) | 237  (78.0%) | 48  (27.3%) | 20  (25.0%) | 113  (78.5%) | 58  (36.3%) | 234  (43.0%) |  |
| Balanced Isotonic Fluid | 1102  (45.0%) | 69  (61.6%) | 55  (20.2%) | 121  (75.6%) | 5  (6.3%) | 123  (76.9%) | 120  (83.3%) | 38  (33.9%) | 43  (14.1%) | 107  (62.2%) | 45  (56.3%) | 17  (11.8%) | 100  (62.5%) | 259  (47.6%) |  |
| Missing answers | 42 | 0 | 19 | 2 | 1 | 1 | 0 | 4 | 6 | 0 | 0 | 0 | 2 | 7 |  |

n = number of centres
