# Supplementary material for: How to follow the guidelines, when the appropriate fluid is missing?
Source: Eur J Pediatr. 2024 Mar 18;183(6):2797–803. doi: 10.1007/s00431-024-05514-6 (PMC11098858; doi:10.1007/s00431-024-05514-6)
Supplement: Supplementary file 2 — Supplementary file2 (DOCX 43 KB) [file 431_2024_5514_MOESM2_ESM.docx]

Supplemental digital content 2: Impact of the availability of balanced fluid on prescription practices

|  | Balanced Isotonic Fluid with dextrose 5% | | Other fluids | | p | Balanced Isotonic Fluid | | Other fluids | | p |
| --- | --- | --- | --- | --- | --- | --- | --- | --- | --- | --- |
|  | n=32 | | n=121 | |  | n=143 | | n=10 | |  |
| Importance of prescribing balanced isotonic fluid (0 to 10 scale) | | | | | | | | | | |
| In conventional unit; median [min-max] | 8 [1-10] | | 7 [0-10] | | 0.98 | 7 [0-10] | | 5.5[0-10] | | 0.82 |
| In critical care unit; median [min-max] | 9 [0-10] | | 9 [0-10] | | 0.87 | 9 [0-10] | | 7.5[1-10] | | 0.77 |
| Proportion of physicians considering balanced solution as a standard of care | | | | | | | | | | |
| Always | 23 | (71,9%) | 42 | (34,7%) | < 0.001 | 63 | (44,1%) | 2 | (20,0%) | 0.19 |
| Limiting factors regarding balanced isotonic fluid prescription | | | | | | | | | | |
| Unavailability of the fluid | 5 | (15,6%) | 40 | (33,1%) | 0.08 | 43 | (30,1%) | 2 | (20,0%) | 0.72 |
| Cost of the fluid | 4 | (12,5%) | 22 | (18,2%) | 0.60 | 24 | (16,8%) | 2 | (20,0%) | 0.68 |
| Ready to use fluid | 12 | (37,5%) | 38 | (31,4%) | 0.53 | 46 | (32,2%) | 4 | (40,0%) | 0.73 |
| Patients characteristics influencing balanced isotonic fluid prescription | | | | | | | | | | |
| Age | 18 | (56,3%) | 43 | (35,5%) | 0.05 | 59 | (41,3%) | 2 | (20,0%) | 0.32 |
| weight | 10 | (31,3%) | 28 | (23,1%) | 0.38 | 37 | (25,9%) | 1 | (10,0%) | 0.44 |
| Patients biological data influencing balanced isotonic fluid prescription | | | | | | | | | | |
| pH | 24 | (75,0%) | 91 | (75,2%) | 1 | 109 | (76,2%) | 6 | (60,0%) | 0.27 |
| Chloremia | 26 | (81,3%) | 93 | (76,9%) | 0.64 | 113 | (79,0%) | 6 | (60,0%) | 0.24 |
| Natremia | 23 | (71,9%) | 73 | (60,3%) | 0.31 | 91 | (63,6%) | 5 | (50,0%) | 0.50 |
| Electrolytes | 14 | (43,8%) | 48 | (39,7%) | 0.69 | 60 | (42,0%) | 2 | (20,0%) | 0.20 |
| Glycemia | 8 | (25,0%) | 42 | (34,7%) | 0.39 | 48 | (33,6%) | 2 | (20,0%) | 0.50 |
| Type of fluids according to clinical situation | | | | | | | | | | |
| *Viral gastroenteritis not tolerating oral rehydration, with normal natremia (137mmol/L); 5 months old* | | | | | | | | | | |
| Unbalanced Hypotonic Fluid | 0 |  | 10 | (8,3%) | 0.0025 | 8 | (5,6%) | 2 | (20,0%) | 0.22 |
| Unbalanced Isotonic Fluid | 8 | (25,0%) | 63 | (52,1%) |  | 66 | (46,2%) | 5 | (50,0%) |  |
| Balanced Isotonic Fluid | 24 | (75,0%) | 47 | (38,8%) |  | 68 | (47,6%) | 3 | (30,0%) |  |
| *Viral gastroenteritis not tolerating oral rehydration, with normal natremia (137mmol/L); 12 years* | | | | | | | | | | |
| Unbalanced Hypotonic Fluid | 0 |  | 8 | (6,6%) | 0.020 | 6 | (4,2%) | 2 | (20,0%) | 0.17 |
| Unbalanced Isotonic Fluid | 9 | (28,1%) | 60 | (49,6%) |  | 65 | (45,5%) | 4 | (40,0%) |  |
| Balanced Isotonic Fluid | 23 | (71,9%) | 52 | (43,0%) |  | 71 | (49,7%) | 4 | (40,0%) |  |
| *Viral gastroenteritis not tolerating oral rehydration, with hypernatremia (> 149 mmol/L); 5 months old* | | | | | | | | | | |
| Unbalanced Hypotonic Fluid | 5 | (15,6%) | 36 | (29,8%) | 0.010 | 38 | (26,6%) | 3 | (30,0%) | 1 |
| Unbalanced Isotonic Fluid | 3 | (9,4%) | 32 | (26,4%) |  | 33 | (23,1%) | 2 | (20,0%) |  |
| Balanced Isotonic Fluid | 24 | (75,0%) | 52 | (43,0%) |  | 71 | (49,7%) | 5 | (50,0%) |  |
| *Viral gastroenteritis not tolerating oral rehydration, with hypernatremia (> 149 mmol/L); 12 years old* | | | | | | | | | | |
| Unbalanced Hypotonic Fluid | 5 | (15,6%) | 33 | (27,3%) | 0.0085 | 35 | (24,5%) | 3 | (30,0%) | 1 |
| Unbalanced Isotonic Fluid | 2 | (6,3%) | 32 | (26,4%) |  | 32 | (22,4%) | 2 | (20,0%) |  |
| Balanced Isotonic Fluid | 25 | (78,1%) | 55 | (45,5%) |  | 75 | (52,4%) | 5 | (50,0%) |  |
| *Status epilepticus with anormal level of consciousness; 5 months old* | | | | | | | | | | |
| Unbalanced Hypotonic Fluid | 0 |  | 6 | (5,0%) | 0.0025 | 5 | (3,5%) | 1 | (10,0%) | 0.51 |
| Unbalanced Isotonic Fluid | 9 | (28,1%) | 75 | (62,0%) |  | 78 | (54,5%) | 6 | (60,0%) |  |
| Balanced Isotonic Fluid | 23 | (71,9%) | 39 | (32,2%) |  | 59 | (41,3%) | 3 | (30,0%) |  |
| *Status epilepticus with anormal level of consciousness; 12 years old* | | | | | | | | | | |
| Unbalanced Hypotonic Fluid | 0 | (0,0%) | 4 | (3,3%) | 0.006 | 3 | (2,1%) | 1 | (10,0%) | 0.31 |
| Unbalanced Isotonic Fluid | 9 | (28,1%) | 71 | (58,7%) |  | 74 | (51,7%) | 6 | (60,0%) |  |
| Balanced Isotonic Fluid | 23 | (71,9%) | 45 | (37,2%) |  | 65 | (45,5%) | 3 | (30,0%) |  |
| *Severe diabetic Keto-acidosis; 6 years old* | | | | | | | | | | |
| Unbalanced Hypotonic Fluid | 1 | (3,1%) | 7 | (5,8%) | 0.40 | 7 | (4,9%) | 1 | (10,0%) | 1 |
| Unbalanced Isotonic Fluid | 14 | (43,8%) | 66 | (54,5%) |  | 75 | (52,4%) | 5 | (50,0%) |  |
| Balanced Isotonic Fluid | 17 | (53,1%) | 46 | (38,0%) |  | 59 | (41,3%) | 4 | (40,0%) |  |
| *Bronchiolitis with persistent respiratory distress under non-invasive ventilatory support; 7 days old* | | | | | | | | | | |
| Unbalanced Hypotonic Fluid | 3 | (9,4%) | 35 | (28,9%) | 0.0005 | 34 | (23,8%) | 4 | (40,0%) | 0.46 |
| Unbalanced Isotonic Fluid | 7 | (21,9%) | 51 | (42,1%) |  | 54 | (37,8%) | 4 | (40,0%) |  |
| Balanced Isotonic Fluid | 22 | (68,8%) | 34 | (28,1%) |  | 54 | (37,8%) | 2 | (20,0%) |  |
| *Bronchiolitis with persistent respiratory distress under non-invasive ventilatory support; 5 months old* | | | | | | | | | | |
| Unbalanced Hypotonic Fluid | 0 |  | 20 | (16,5%) | 0.0005 | 17 | (11,9%) | 3 | (30,0%) | 0.22 |
| Unbalanced Isotonic Fluid | 8 | (25,0%) | 63 | (52,1%) |  | 66 | (46,2%) | 5 | (50,0%) |  |
| Balanced Isotonic Fluid | 24 | (75,0%) | 37 | (30,6%) |  | 59 | (41,3%) | 2 | (20,0%) |  |
| *Pneumonia with persistent respiratory distress under non-invasive ventilatory support; 12 years old* | | | | | | | | | | |
| Unbalanced Hypotonic Fluid | 0 |  | 13 | (10,7%) | 0.0005 | 11 | (7,7%) | 2 | (20,0%) | 0.38 |
| Unbalanced Isotonic Fluid | 7 | (21,9%) | 63 | (52,1%) |  | 65 | (45,5%) | 5 | (50,0%) |  |
| Balanced Isotonic Fluid | 25 | (78,1%) | 44 | (36,4%) |  | 66 | (46,2%) | 3 | (30,0%) |  |
| *24 hours post-appendectomy monitoring, nil by mouth; 5 months old* | | | | | | | | | | |
| Unbalanced Hypotonic Fluid | 0 |  | 16 | (13,2%) | 0.0005 | 14 | (9,8%) | 2 | (20,0%) | 0.55 |
| Unbalanced Isotonic Fluid | 6 | (18,8%) | 57 | (47,1%) |  | 58 | (40,6%) | 5 | (50,0%) |  |
| Balanced Isotonic Fluid | 26 | (81,3%) | 44 | (36,4%) |  | 67 | (46,9%) | 3 | (30,0%) |  |
| *24 hours post-appendectomy monitoring, nil by mouth; 12 years old* | | | | | | | | | | |
| Unbalanced Hypotonic Fluid | 0 |  | 11 | (9,1%) | 0.0085 | 9 | (6,3%) | 2 | (20,0%) | 0.35 |
| Unbalanced Isotonic Fluid | 7 | (21,9%) | 56 | (46,3%) |  | 58 | (40,6%) | 5 | (50,0%) |  |
| Balanced Isotonic Fluid | 25 | (78,1%) | 51 | (42,1%) |  | 73 | (51,0%) | 3 | (30,0%) |  |
| *Acute Respiratory Distress Syndrome under invasive ventilatory support; 5 months old* | | | | | | | | | | |
| Unbalanced Hypotonic Fluid | 0 |  | 14 | (11,6%) | 0.001 | 12 | (8,4%) | 2 | (20,0%) | 0.41 |
| Unbalanced Isotonic Fluid | 7 | (21,9%) | 62 | (51,2%) |  | 66 | (46,2%) | 3 | (30,0%) |  |
| Balanced Isotonic Fluid | 25 | (78,1%) | 44 | (36,4%) |  | 64 | (44,8%) | 5 | (50,0%) |  |
| *Acute Respiratory Distress Syndrome under invasive ventilatory support; 12 years old* | | | | | | | | | | |
| Unbalanced Hypotonic Fluid | 0 |  | 7 | (5,8%) | 0.0045 | 6 | (4,2%) | 1 | (10,0%) | 0.86 |
| Unbalanced Isotonic Fluid | 7 | (21,9%) | 63 | (52,1%) |  | 66 | (46,2%) | 4 | (40,0%) |  |
| Balanced Isotonic Fluid | 25 | (78,1%) | 50 | (41,3%) |  | 70 | (49,0%) | 5 | (50,0%) |  |
| *Post-traumatic brain injury with raised intra-cranial pressure; 14 years old* | | | | | | | | | | |
| Unbalanced Hypotonic Fluid | 0 |  | 2 | (1,7%) | 0.14 | 2 | (1,4%) | 0 | (0,0%) | 0.83 |
| Unbalanced Isotonic Fluid | 12 | (37,5%) | 69 | (57,0%) |  | 76 | (53,1%) | 5 | (50,0%) |  |
| Balanced Isotonic Fluid | 19 | (59,4%) | 47 | (38,8%) |  | 61 | (42,7%) | 5 | (50,0%) |  |
| *Post-cardiac surgery with bypass; 5 months old* | | | | | | | | | | |
| Unbalanced Hypotonic Fluid | 1 | (3,1%) | 19 | (15,7%) | 0.001 | 18 | (12,6%) | 2 | (20,0%) | 0.67 |
| Unbalanced Isotonic Fluid | 3 | (9,4%) | 47 | (38,8%) |  | 48 | (33,6%) | 2 | (20,0%) |  |
| Balanced Isotonic Fluid | 23 | (71,9%) | 42 | (34,7%) |  | 61 | (42,7%) | 4 | (40,0%) |  |
| *All situations* | | | | | | | | | | |
| Unbalanced Hypotonic Fluid | 15 | (2,9%) | 241 | (12,4%) | < 0.001 | 225 | (9,8%) | 31 | (19,4%) | 0.0025 |
| Unbalanced Isotonic Fluid | 118 | (23,0%) | 930 | (48,0%) |  | 980 | (42,8%) | 68 | (42,5%) |  |
| Balanced Isotonic Fluid | 373 | (72,9%) | 729 | (37,7%) |  | 1043 | (45,6%) | 59 | (36,9%) |  |
| Missing data | 6 |  | 36 |  |  | 40 |  | 2 |  |  |

n = number of centres
